# Paxlovid reduces the 28-day mortality of patients with COVID-19: a retrospective cohort study

**DOI:** 10.1186/s12879-024-09482-y

**Published:** 2024-08-01

**Authors:** Kaican Zong, Li Xu, Chun Luo, Chen Luo, Bin Liu, Jiacheng Chen, Huizi Wu, Zhiqiang Liu, Rongjuan Zhuang, Shuliang Guo

**Affiliations:** 1 Department of Respiratory and Critical Care Medicine, The First Affiliated Hospital, Chongqing Medical University, Chongqing, 400016 People’s Republic of China; 2 Department of Respiratory and Critical Care Medicine, Affiliated University Town Hospital of Chongqing Medical University, Chongqing, 401331 People’s Republic of China; 3 Department of Respiratory and Critical Care Medicine, The Seventh People’s Hospital of Chongqing (Affiliated Central Hospital of Chongqing University of Technology), Chongqing, 400054 People’s Republic of China

**Keywords:** Paxlovid, SARS-CoV-2, COVID-19, Mortality, Treatment

## Abstract

**Purpose:**

In this study, we aim to explore the efficacy of paxlovid on reducing mortality of COVID-19 patients in clinical setting, especially whether paxlovid modifies the risk of death in these severe and critical patients.

**Methods:**

Our retrospective cohort study was conducted on the medical records of patients, consecutively admitted for COVID-19 to five hospitals in Chongqing, China from Dec 8, 2022 to Jan 20, 2023. Based on whether patients received paxlovid during their hospitalization, patients were grouped as paxlovid group and non-paxlovid group. We used 1:1 ratio propensity score matching (PSM) in our study to adjust for confounding factors and differences between groups. Statistical analysis were performed by SPSS 23.0. The differences in 28-day mortality between these two groups and its influencing factors were the main results we focused on.

**Results:**

There were 1018 patients included in our study cohort. With 1:1 ratio PSM, each of the paxlovid group and non-paxlovid group included 237 patients. The results showed that patients using paxlovid have a lower 28-day mortality in overall population either before PSM (OR 0.594, 95% CI 0.385–0.917, *p* = 0.019) or after PSM (OR 0.458, 95% CI 0.272–0.774, *p* = 0.003) with multivariable adjusted logistic regression models. Meanwhile, in severe subgroup, it showed similar findings.With paxlovid treatment, it showed a significantly lower 28-day mortality in severe subgroup both before PSM (28% vs.41%, *p* = 0.008) and after PSM (19% vs.32%, *p* = 0.007).

**Conclusion:**

Paxlovid can significantly reduce the risk of 28-day mortality in overall population and severe subgroup patients.This study distinguished the severe subgroup patients with COVID-19 who benefit more from paxlovid treatment.

## Introduction

Coronavirus disease of 2019 (COVID-19), caused by severe acute respiratory syndrome coronavirus 2 (SARS-CoV-2), poses a huge burden owing to its rapid transmission [1]. As of Feb 12, 2023, there have been confirmed more than 757 million cases, and it has resulted more than 6 million deaths [2]. Human scientists and healthcare workers have dedicated to research how to prevent SARS-CoV-2 infection and treat patients in order to achieve the goal of reducing infections and deaths. Fortunately, the treatment options for COVID-19 have significantly increased in recent years [3, 4]. In clinical practice, there may be multiple risk factors for SARS-CoV-2 infection, including vaccination, underlying disease conditions, severity of illness, etc. In the study of Ghasemi D et al. [5], they found that anti-SARS-CoV-2 antibodies in the blood can persist for 6 months post-infection, it may be against SARS-CoV-2 infection for some time. However their levels steeply declined over time. Hosseinzadeh R et al [6] pointed out that COVID-19 patients with hypertensive, age > 60 years, BMI > 25Kg/m^2^, diabetes and chronic kidney disease are associated with poor outcomes. And predictors for severity of SARS-CoV-2 infection also include underlying liver diseases [7].

At the same time, the severity and prognosis of the disease caused by SARS-CoV-2 infection is also related to the patient’s underlying disease situation. Therefore, it may be very important to choose a personalized treatment for different patients with COVID-19. Pontolillo M et al [8] reported their experience in the use of molnupiravir. They pointed out that patients receiving molnupiravir exhibited advantages such as early clinical improvement, no need for hospitalization, and low incidence of adverse events. Saravolatz LD et al [9] reviewed the antiviral activity of paxlovid and molnupiravir, which included these 2 oral COVID-19 antiviral drugs’ mechanisms of action, clinical experience, recommended indications, etc. Ucciferri C et al [10] found that no difference of therapeutic response was observed in patients treated with monoclonal antibody, whether they were obese or not. And they also found that pidotimod can promote faster virological recovery as an effective, low-cost drug in the first phase of disease [11]. Nejat N et al [12] reminded people that some traditional methods for COVID-19 prevention and treatment may lack the necessary effectiveness and have side effects. And they gave the suggestion that necessary training should be provided to the public about using treatment for COVID-19. These studies tell us that the treatment experience for COVID-19 needs to sum up repeatedly in clinical practice, which may lead to effective therapeutic options. As a potential Mpro inhibitor, paxlovid shows good antiviral activity in vivo and has been approved by the National Medical Products Administration for emergency treatment of COVID-19 [13]. It is an oral antiviral drug produced by Pfizer for using against COVID-19 [14]. Many advances have been made in development of anti-SARS-CoV-2 inhibitors, and the efficacy for mild and moderate COVID-19 patients has been proven [15, 16]. But some important outcome indicators such as 28-day mortality were rarely studied. There were also some important problems to use the anti-SARS-CoV-2 therapeutics that need to be explored, such as whether paxlovid also benefit severe and critical patients with COVID-19. In this study, we aim to explore the efficacy of paxlovid on reducing mortality of COVID-19 patients in clinical setting, especially whether paxlovid modifies the risk of death in these severe and critical patients. We would like to provide our clinical experiences in using paxlovid to treat COVID-19 patients.

## Patients and methods

Our study was conducted on the medical information of patients, consecutively admitted for COVID-19 to five hospitals in Chongqing from Dec 8, 2022 to Jan 20, 2023. The inclusion criteria are as follows: (1) nasopharyngeal swab specimens that confirmed SARS-CoV-2 nucleic acid positivity by RT-PCR. (2) the diagnostic criteria for COVID-19 was accorded with “Diagnosis and Treatment Program for Novel Coronavirus Pneumonia (tenth Edition)” which published by the National Health Commission and National Administration of Traditional Chinese Medicine^17^. (3) age ≥ 18years. (4) Female were not pregnant or breastfeeding. Exclusion criteria: (1) using azvudine for anti-SARS-CoV-2 (During the research period, only azvudine and paxlovid were the available antiviral drugs ). (2) patients with immunodeficiency or immunosuppression. The study was approved by the Institutional Research Ethics Committee of the First Affiliated Hospital of Chongqing Medical University (Chongqing, China). The study was performed in accordance with relevant guidelines and regulations. Records were extracted by three of the authors from the medical records systems of each hospital, and the lead author verified the data accuracy. Baseline data were collected on the first day of hospital admission which included age, gender, medical history, pre-existing comorbid conditions, COVID-19 testing results, clinic type, laboratory test results at admission, the use of systemic corticosteroids, budesonide, paxlovid, supplemental oxygen and invasive or noninvasive mechanical ventilation support. Based on whether patients received Paxlovid at any point during their hospitalization, patients were grouped as follows: paxlovid group and non-paxlovid group. At present, patients with COVID-19 were classified into four grades (mild, moderate, severe and critical) by “Diagnosis and Treatment Program for Novel Coronavirus Pneumonia (tenth Edition)” which published by the National Health Commission and National Administration of Traditional Chinese Medicine [17]. In our study, these patients were classified into moderate patients and severe sub-group (including severe and critical patients) according to the standard.

In this study, primary outcome was 28-day mortality that was from the date of admission. Mortality data for discharged patients was gathered from telephone follow-ups.

### Statistical analysis

SPSS 23.0, the R package (version 4.2.2), and PSM plug-ins (version 3.0.4) were used for the statistical analysis. For the parameters with a Gaussian distribution, Data were expressed as means ± standard deviations (mean ± SD). And for the parameters with a non-Gaussian distribution, they were expressed as medians and range (median ± IQR). Comparisons were made using parametric (Student’s) or non-parametric (Mann–Whitney U) tests. Chi-square test was used for categorical variables. It was considered statistically significant if *p*-values was less than 0.05. The association between risk factors and 28-day mortality were used logistic regression models to caculate the adjusted odds ratios (ORs) and its 95% CIs. We used 1:1 ratio propensity score matching (PSM) in our study to adjust for confounding factors and differences between groups. The paxlovid group was matched with the non-paxlovid group without replacement according to the propensity scores generated. For PSM, we set a caliper value of 0.15. Patients’ baseline between the Paxlovid group and the non-Paxlovid group was matched by using nearest-neighbor matching. And the standardized mean difference (SMD) was computed before and after matching in order to determine whether the PSM lessened the differences in pretreatment covariates between the paxlovid and non-paxlovid groups. Finally, we included potential confounding factors of clinical expertise judgment as well as variables with *p* < 0.1 and used the Cox model and multivariate logistic regression model to adjust the residual imbalance.

## Results

**Fig. 1:**
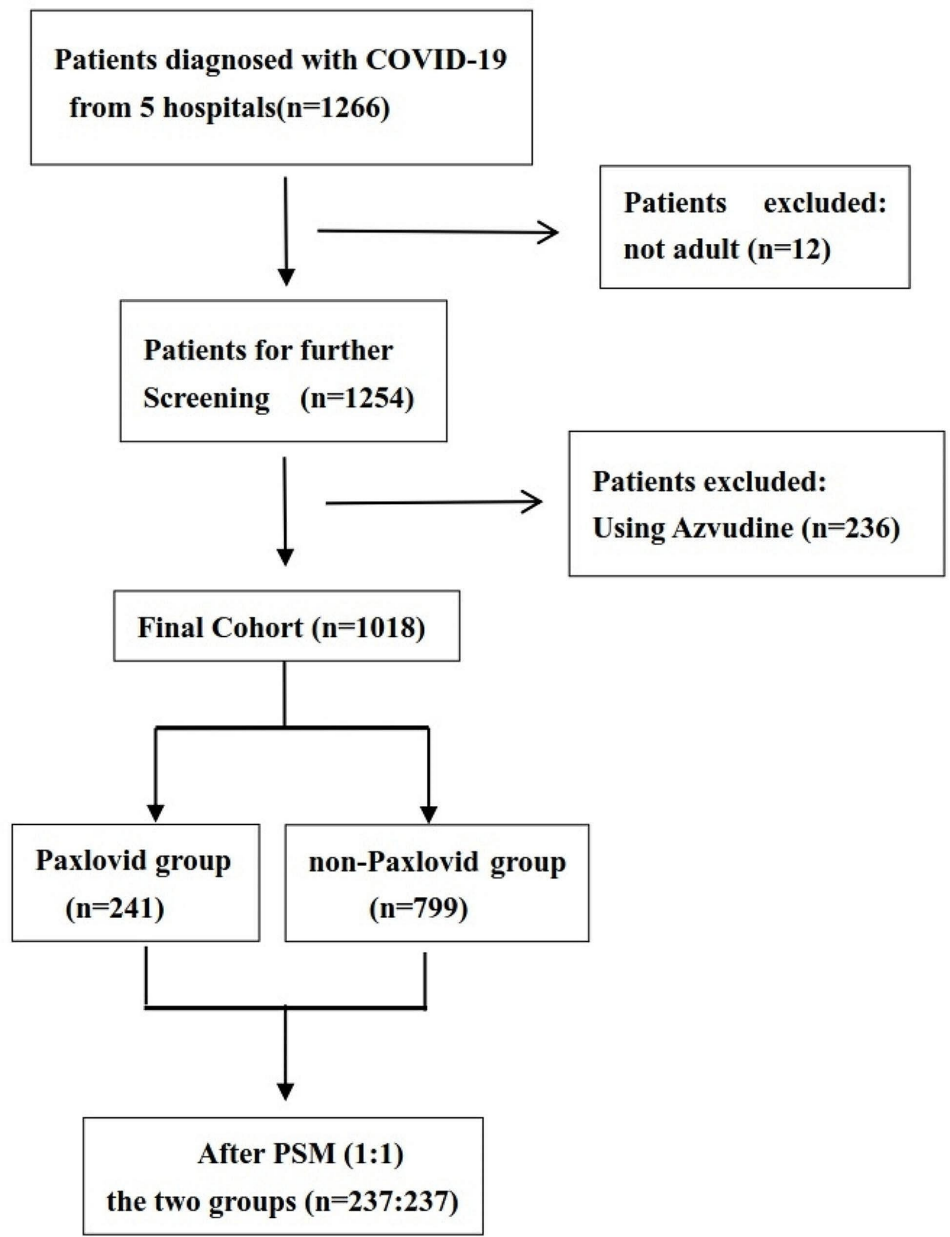
Flowchart of patient selection

COVID-19 patients admitted to the hospital during the study period were 1266. Of them, 12 patients younger than 18 years and 236 patients used azvudine were excluded. Finally, 1018 patients were included in our study cohort. In general, there were 241 patients received paxlovid treatment during hospitalization, while 777 patients did not expose to paxlovid treatment. With 1:1 ratio PSM, each of the paxlovid group and non-paxlovid group included 237 patients (Fig. 1).

### Baseline characteristics of included patients

In original and matched cohort, the clinical characteristics of the paxlovid group and the non-paxlovid group were shown in Table 1. Before PSM, there were significant differences in frequency of Chronic pulmonary disease (12% vs.18%, *p* = 0.046), Chronic liver disease (2% vs.6%, *p* = 0.024), CRP (41.2 vs.13.93, *p* = 0.000), P/F (267 vs.276.7, *p* = 0.011), and Hospital stay (9.68 vs.8.63, *p* = 0.011) between paxlovid group and non-paxlovid group. In matched cohort, it showed no significant differences in these aspects.

**Table 1:** The demographic and clinical characteristics of COVID-19 patients with Paxlovid treatment or not

	Original cohort				Matched cohort			
	Paxlovid (n = 241)	non-Paxlovid (n = 777)	*P* value	SMD	Paxlovid (n = 237)	non-Paxlovid (n = 237)	*P* value	SMD
Age, years(mean ± SD) Gender,male Diabetes Cardiovascular diseases Chronic pulmonary disease Chronic kidney disease Chronic liver disease	71.14 ± 14.95 151(75%) 66(27%) 99(41%) 29(12%) 36(15%) 5(2%)	69.7 ± 15.16 453(58%) 217(28%) 365(47%) 137(18%) 102(13%) 43(6%)	**P = 0.196** **P = 0.260** **P = 0.934** **P = 0.120** **P = 0.046** **P = 0.518** **P = 0.024**	0.096 -0.09 -0.012 -0.12 -0.172 0.051 -0.242	71.0 ± 15.036 148(62%) 64(27%) 97(41%) 29(12%) 36(15%) 5(2%)	71.45 ± 14.063 157(66%) 67(28%) 100(42%) 23(10%) 35(15%) 6(3%)	**P = 0.738** **P = 0.443** **P = 0.837** **P = 0.852** **P = 0.463** **P = 1.000** **P = 1.000**	-0.030 0.078 -0.028 -0.026 0.078 0.012 -0.030
**Laboratory tests**								
Neutrophil (median ± IQR)×10⁹ Lymphocyte (mean ± SD)×10⁹ Platelet (median ± IQR)×10⁹ CRP (median ± IQR) – mg/dL PCT (median ± IQR) – ng/mL DD (median ± IQR) – ng/mL P/F (median ± IQR) Moderate subgroup Severe subgroup	5.02(3.38–7.64) 0.87 ± 0.55 157(116–211) 41.2(12.7–90) 0.15(0.07–0.8) 1.06(0.48–2.36) 253(168-329.25) 81(34%) 160(66%)	4.73(2.93–7.05) 0.96 ± 0.64 166(123–227) 13.93(3.45-59) 0.14(0.07–0.53) 0.88(0.47–1.91) 283.5(203–376) 293(38%) 484(62%)	**P = 0.086** **P = 0.060** **P = 0.193** **P = 0.000** **P = 0.416** **P = 0.238** **P = 0.011** **P = 0.253** **P = 0.253**	0.063 -0.141 -0.098 0.405 0.037 -0.327 -0.134 -0.087 -0.107	5.02(3.37–7.81) 0.88 ± 0.55 161(117–214) 41.5(11.8–90) 0.13(0.07–0.69) 0.99(0.47–2.21) 267(188–364) 79(33%) 158(67%)	5.25(3.47–7.83) 0.86 ± 0.67 156(118.5-214.5) 29.9(6-97.25) 0.18(0.07–0.92) 0.87(0.48–2.28) 276.7(197–373) 76(32%) 161(68%)	**P = 0.658** **P = 0.696** **P = 0.600** **P = 0.070** **P = 0.405** **P = 0.949** **P = 0.893** **P = 0.952 P=0.952**	0.045 0.040 0.032 0.068 0.011 -0.025 0.044 0.027 -0.025
**Outcomes**								
Hospital stay, days (mean ± SD) 28-day mortality	9.68 ± 5.29 44(18%)	8.63 ± 5.67 188(24%)	**P = 0.011** **P = 0.065**		9.7 ± 5.27 43(18%)	9.34 ± 6.43 62(26%)	**P = 0.502** **P = 0.046**	

**Definition of abbreviations:** SD (standard deviation), SMD (standardized mean difference), IQR (interquartile range), CRP (C-reactive protein), PCT (procalcitonin), DD (D-dimer), P/F (Oxygenation index, PaO2/FiO2 ration, ratio of partial pressure of O2 in arterial blood to fraction of inspired oxygen).

### Primary outcomes

We analyzed the association of using paxlovid and 28-day mortality of COVID-19 patients in overall and subgroups (Table 2). The results showed that using paxlovid was associated with reduced 28-day mortality (OR 0.626, 95% CI 0.403–0.971, *p* = 0.036) after PSM. With multivariable adjusted logistic regression models, the results showed that patients using paxlovid have a lower mortality in overall population either before PSM (OR 0.594, 95% CI 0.385–0.917, *p* = 0.019) or after PSM (OR 0.458, 95% CI 0.272–0.774, *p* = 0.003). Meanwhile, in severe subgroup, the results showed that patients using paxlovid have a lower 28-day mortality both with univariate analysis (OR 0.491, 95% CI 0.293–0.824, *p* = 0.007) and multivariable analysis (OR 0.413, 95% CI 0.223–0.765, *p* = 0.005) after PSM.

**Table 2:** Association of using Paxlovid and 28-day mortality of COVID-19 patients in overall and subgroups

	Univariate analysis			Multivariate analysis		
28- **day mortality**	**β-value**	**OR(95%CI)**	***P***-value	**β-value**	**OR(95%CI)**	***P***-value
Overall patients before PSM (*n* = 1018) Overall patients after PSM (*n* = 474, match 1:1) Moderate subgroup patients after PSM (*n* = 155) Severe subgroup patients after PSM (*n* = 319)	0.166 -0.469 0.262 -0.711	1.181(0.823 to 1.694) 0.626(0.403 to 0.971) 1.300(0.533 to 3.173) 0.491(0.293 to 0.824)	*P* = 0.367 *P* = 0.036 *P* = 0.564 *P* = 0.007	−0.520 −0.780 −0.278 -0.884	0.594(0.385 to 0.917) 0.458(0.272 to 0.774) 0.757(0.215 to 2.670) 0.413(0.223 to 0.765)	*P* = 0.019 *P* = 0.003 *P* = 0.665 *P* = 0.005

**Definition of abbreviations:** PSM (propensity score matching), OR (adjusted odds ratio), CI (confidence interval).

**Fig. 2:**
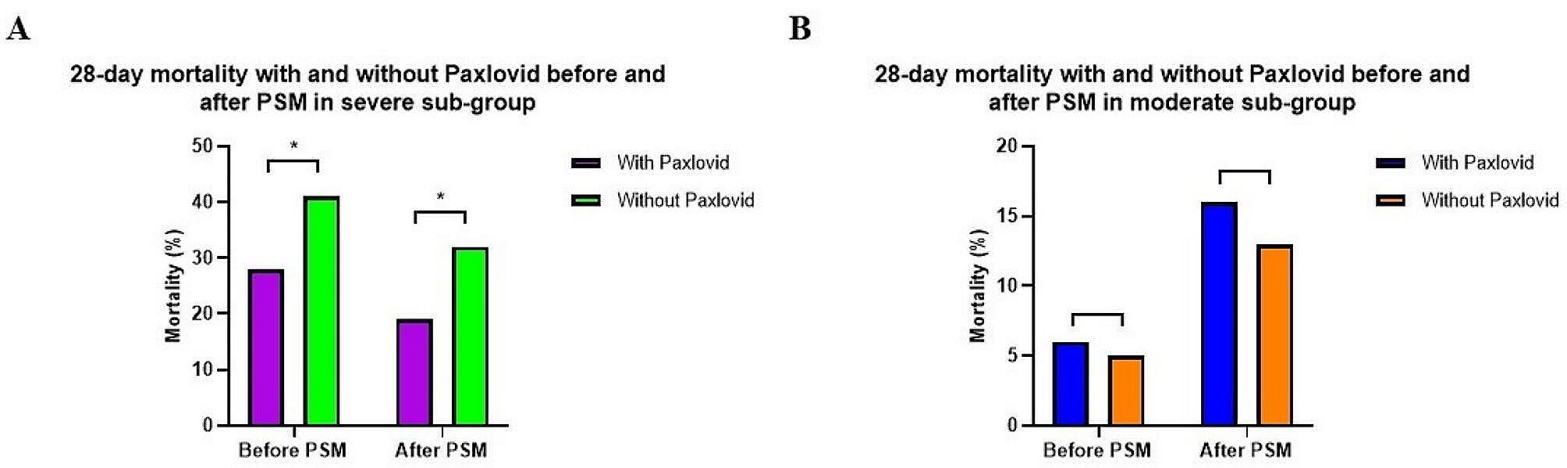
**(A and B)**: Association between Paxlovid treatment and 28-day mortality of COVID-19 patients in each subgroup

Furthermore, analysis association between paxlovid treatment and 28-day mortality of COVID-19 patients in each subgroup before and after PSM was shown in Fig. 2. With paxlovid treatment, it showed a significantly lower 28-day mortality in severe subgroup both before PSM (28% vs.41%, *p* = 0.008) and after PSM (19% vs.32%, *p* = 0.007).

### Risk factors of 28-day mortality

Finally, we estimated the risk factors associated with higher 28-day mortality in matched cohort (Table 3). We found that the risk factors associated with higher 28-day mortality could be older age (OR 1.040, 95%CI 1.019 to 1.062, *P*= 0.000), Diabetes (OR 1.863, 95%CI 1.063 to 3.265, *P*= 0.030), Cardiovascular diseases (OR 1.737, 95%CI 1.021 to 2.952, *P*= 0.041), higher Neutrophil count (OR 1.120, 95%CI 1.041 to 1.206, *P*= 0.003), and lower oxygenation index (OR 0.996, 95%CI 0.994 to 0.998, *P*= 0.001). The COVID-19 patients received supplemental oxygen (OR 3.332, 95%CI 1.484 to 7.485, *P*= 0.004) and NIV (OR 3.033, 95%CI 1.749 to 5.261, *P*= 0.000) treatment also had a higher 28-day mortality.

**Table 3:** Factors related to 28-day mortality of COVID-19 patients with logistic regression model after PSM

Variables	OR	Lower.95	Upper.95	*P*-value
Age Diabetes Cardiovascular diseases Chronic kidney disease Neutrophil Lymphocyte C-reactive protein(CRP) Oxygenation index Systemic corticosteroids Supplemental oxygen NIV IMV	1.040 1.863 1.737 1.388 1.120 0.792 1.001 0.996 0.976 3.332 3.033 1.785	1.019 1.063 1.021 0.695 1.041 0.515 0.997 0.994 0.537 1.484 1.749 0.904	1.062 3.265 2.952 2.769 1.206 1.217 1.006 0.998 1.771 7.485 5.261 3.523	*P* = 0.000 *P* = 0.030 *P* = 0.041 *P* = 0.353 *P* = 0.003 *P* = 0.288 *P* = 0.472 *P* = 0.001 *P* = 0.935 *P* = 0.004 *P* = 0.000 *P* = 0.095

**Definition of abbreviations:** PSM (propensity score matching), OR (adjusted odds ratio), NIV(Non-invasive mechanical ventilation), IMV(Invasive mechanical ventilation).

## Discussion

In our study, we analyzed the association of paxlovid using and 28-day mortality of COVID-19 patients in overall and subgroups before and after PSM. We found that paxlovid treatment reduced 28-day mortality of COVID-19 patients in severe subgroup from 41 to 28% before PSM and from 32 to 19% after PSM. Meanwhile, the result showed that using paxlovid was associated with a higher mortality in overall and severe subgroup population, both with univariate analysis and multivariable adjusted logistic regression models. Many other studies focused on the efficacy of paxlovid in COVID-19. Paxlovid is a new oral antiviral drug, produced by Pfizer to use against COVID-19, given for 5 consecutive days to patients with mild to moderate disease [18, 19]. In another study [20], half of the participants received paxlovid and the other half received placebo administered orally every 12 h for five consecutive days. The study revealed that paxlovid was 89% effective in patients at risk of serious illness. In a meta-analysis [21], the study shows that paxlovid is effective in reducing the mortality and hospitalization rates in COVID-19 patients. In addition, the study showed that the paxlovid did not increase the occurrence of adverse events, thus exhibiting good overall safety. Najjar-Debbiny R et al [22] pointed out that both paxlovid and adequate COVID-19 vaccination status were associated with significant decrease in the rate of severe COVID-19 or mortality. These studies have found on the efficacy of paxlovid in COVID-19 patients and their research findings are consistent with ours in many aspects.

However, some other studies have to some extent expressed concerns about the use of paxlovid. Arbel R et al [23] reported that paxlovid significantly reduce the hospitalization and death rates in patients over 65 years old but not those under 65 years. Najjar-Debbiny R et al [22] also pointed out paxlovid appears to be more effective in older patients. It seems that it is very important to choose a personalized therapeutic plan according to the basic condition and severity of COVID-19 patients.

During our study period, there were only two antiviral drugs available in our city, so some patients with COVID-19 were treated with paxlovid. In our study, we conducted a subgroup analysis on COVID-19 with different severity.We found paxlovid treatment significantly reduced 28-day mortality of COVID-19 patients in severe subgroup. Our findings were validated after multivariate adjustment for differences and comorbidities between the treatment and control group, and also in a PSM cohort. We believe that this result is reliable and consistent with the actual situation observed in our clinical practice.

This study has some limitations. First, it is a retrospective study. This is mainly a real-life database. Due to the accessibility of drugs, our choice of antiviral therapy is limited. We can not assess the effect of other important variables. Second, we mainly concerned about the impact of paxlovid on mortality, while had not strictly ruled out the role of traditional Chinese medicine. Third, a large number of patients not admitting to hospital were excluded, which made this study unable to evaluate the efficacy of paxlovid in these patients. Although this study has several limitations, it still provides good evidences about the role of paxlovid in patients with COVID-19.

## Conclusions

In conclusion, paxlovid can significantly reduce the risk of 28-day mortality in overall population and severe subgroup patients with COVID-19. This is a very meaningful discovery, because it validated the effect of paxlovid treating patients with COVID-19 in the clinical setting. More importantly, this study distinguished the severe subgroup patients with COVID-19 who benefit more from paxlovid treatment. If our study results were confirmed by more clinical trials, it will increase the treatment options of patients with severe COVID-19.

## Data Availability

The data that support the findings of this study are available on request from the corresponding author.
